# Perception and knowledge of dementia prevention and its associated socio-demographic factors in China: A community-based cross-sectional study

**DOI:** 10.3389/fnins.2022.1093169

**Published:** 2022-12-05

**Authors:** Dan Song, Doris Yu, Qiuhua Sun

**Affiliations:** ^1^Department of Nursing, Shenzhen Qianhai Shekou Free Trade Zone Hospital, Shenzhen, China; ^2^School of Nursing, The University of Hong Kong, Hong Kong, Hong Kong SAR, China; ^3^School of Nursing, Zhejiang Chinese Medical University, Hangzhou, China

**Keywords:** dementia, risk factors, prevention, belief, knowledge

## Abstract

**Background:**

Although considerable progress has been made on the risk factors of dementia, less is known about the extent of the gaps between the general public’s understanding of dementia prevention and contemporary scientific evidence. This study aimed to determine the beliefs and knowledge of dementia prevention among the Chinese general public and examine the socio-demographic factors of the belief and knowledge of dementia prevention.

**Methods:**

The study adopted a cross-sectional design. A total of 358 Chinese adults aged over 40 years were recruited from four healthcare centers. We designed questionnaires that include items on the belief of dementia prevention, risk factors for dementia, and health education needs regarding dementia prevention based on previous literature. Descriptive statistics and multivariate regression analyses were conducted.

**Results:**

Only 32.4% of the respondents agreed that dementia is preventable. Less participants were able to correctly identify cardiovascular risk factors (i.e., obesity, diabetes, dyslipidemia, hypertension, unhealthy diet, smoking, and alcohol) as part of dementia risk factors. Younger age, higher education, and having contact with patients with dementia are associated with stronger belief that dementia is preventable. Older age, higher income, higher education, having memory complaint, and having contact with patients with dementia are associated with a better understanding of dementia risk factors. A total of 88.9% respondents thought that they are not well informed of dementia from public education, and most respondents (65%) prefer receiving dementia-related health advice from primary care providers.

**Conclusion:**

The present study reveals the great gaps between the Chinese general public’s knowledge of dementia prevention and the latest research evidence. Public health educational programs for all age groups are encouraged to close this knowledge gap. More attention and resources should be paid to individuals with low income and low education level as they have limited access to dementia prevention information. Researchers should work in partnership with primary care providers to help translate evidence into community practice with a special focus on the link between cardiovascular risk factors and dementia.

## Introduction

The rapid aging of the global population entails an increase in the prevalence of cognitive impairment among older adults (Bettio et al., 2017). Dementia is one of the principal causes of disability and decreased quality of life amongst the elderly. In 2020, over 50 million people worldwide are living with Dementia (World Health Organization [WHO], 2022). This number is expected to increase to 63 million in 2030 and 114 million in 2050 (Wu et al., 2017). By 2030, the annual cost for dementia globally is projected to be 2 trillion dollars (Prince et al., 2016). Dementia has reached the forefront of the public health agenda because of its tremendous physical, emotional, and economic burden.

Dementia has no cure, but has a wide range of potentially modifiable risk factors. In 2019, the World Health Organization (WHO) published the latest guideline on the risk reduction of cognitive decline and dementia (World Health Organization [WHO], 2019). This guideline reviewed existing evidence on the 12 most substantial modifiable risk factors for dementia, including cardiovascular risk factors (i.e., hypertension, diabetes, dyslipidemia, and obesity), psycho-social factors (i.e., depression and social isolation), and lifestyle factors (i.e., low level of physical or mental activity, unbalanced diet, alcohol abuse and smoking, and hearing loss). Roughly one third of all dementia cases could be potentially prevented through the management of the modifiable risk factors (Livingston et al., 2017). These evidences highlight the opportunity for dementia prevention.

A good understanding of the modifiable risk factors for dementia may encourage preventative health behaviors, which ultimately reduce the incidence of dementia. In 2017, WHO released the global action plan on dementia, which urges all countries to implement campaigns to raise public awareness about dementia (World Health Organization [WHO], 2017). One of the major priorities to inform this action is to determine the knowledge gaps about cognitive health and related risk factors among the general public such that education programs can be most effectively targeted.

Although researchers have made considerable progress on the risk and preventive factors of dementia, less is known about the extent of the gaps between the general public’s understanding of dementia prevention and contemporary scientific evidence. Yeo et al. (2007) surveyed older adults in the UK and found a poor overall knowledge of the risk and protective factors for dementia. Connell et al. (2007) surveyed the general public in the USA and found that nearly half of the respondents see dementia as unpreventable. Low and Anstey examined the patterns of beliefs underlying the behaviors and beliefs of the Australian public on what can reduce dementia risk (Low and Anstey, 2009); they concluded that the public perceptions of what might reduce dementia risk are not influenced by scientific evidence. The beliefs and knowledge about dementia prevention of those living in low- and middle-income countries are still largely unknown. China has the largest aging population and faces tremendous burden on dementia care. The general public’s knowledge of dementia prevention is necessary to understand first to inform dementia risk reduction public health campaigns.

Therefore, the aim of this study was to compare the Chinese general public’s understanding of dementia prevention and contemporary scientific evidence and to identify the socio-demographic factors related to the beliefs and level of knowledge regarding dementia prevention. Such an assessment can provide important insights for the design of dementia risk reduction strategies.

## Materials and methods

### Design and sample

A cross-sectional study design was used. This study was conducted in four community healthcare centers (CHCs) in Hangzhou City, southeast China, including Tianshui CHC, Wulin CHC, Huanbei CHC, and Huanxi CHC. In China, CHCs are set up by the government in communities to provide basic medical and public health services for the communities residents. The main work of medical treatment service in CHCs is to consult, diagnose, and treat residents for the common and frequently occurring chronic diseases. A consecutive sample of 358 adults aged over 40 years who visited the CHCs for general medical service from September 2019 to December 2019 was recruited. Participants were excluded if they had a diagnosis of dementia or cognitive impairment or had impaired hearing or vision, which may inhibit them from giving consent and answering the questionnaires. This study was approved by the Zhejiang Chinese Medical University (No. SBREC-20181231). Written consent was obtained from each participant. All information was kept strictly confidential.

### Measures

The questionnaire was designed by the research team in partnership with a neurological physician based on the most current evidence. Standardized tests, such as the Alzheimer’s Disease Knowledge Scale (Carpenter et al., 2009), were not appropriate for this study, as they include few questions on dementia risk factors and do not have dementia prevention sub-scales according to the latest evidence. The self-designed questionnaire (Supplementary material) includes items on beliefs of dementia prevention, risk factors for dementia, and health education needs regarding dementia.

### Attitude toward dementia prevention

The first section of the questionnaire contains two questions. The respondents were asked to circle 1 of 3 options: “yes,” “no,” and “no idea.” The questions include (1) “Do you think that dementia is caused by normal aging?” (2) “Do you think that the risk of dementia can be reduced?”

### Risk factors for dementia

The second section of the questionnaire has 12 items on dementia risk factors, which were chosen based on the latest evidence reported by the 2019 WHO guidelines on risk reduction of cognitive decline and dementia (World Health Organization [WHO], 2019). The dementia risk factors include hearing loss, smoking, alcohol abuse, unbalanced and unhealthy diet, hypertension, diabetes, dyslipidemia, obesity, physical inactivity, cognitive inactivity, depression, and social isolation. Participants were asked to identify risk factors and circle 1 of 3 options: “yes,” “no,” and “no idea.”

### Health education needs regarding dementia prevention

The last section of the questionnaire includes one item that asks the respondents whether they are well informed of dementia prevention from public education and five items regarding their preferred health education delivery format. The education delivery format includes community bulletin board, health talks by experts, advice from family physicians and community nurses, education booklets, and regular peer sharing. Participants were asked to tick their preferred education content and delivery format in the questionnaire.

### Socio-demographic information

The key socio-demographic factors include age, gender, education, and income. In addition, the respondents were also asked to indicate whether they had memory complaint and whether they were in contact with anyone who had dementia.

### Statistical analyses

Statistical analysis was performed with SPSS version 22. Continuous data are summarized in means with standard deviation (SD), and categorical data are presented as count and percentage. We calculated the percentage of participants who correctly answered each item on the questionnaire to assess the dementia prevention beliefs and knowledge. Multiple logistic regression was conducted with each item regressed on the socio-demographic factors. Adjusted odds ratios (AORs) and 95% confidence intervals (CIs) are presented. A Bonferroni multiple comparison correction was applied to the results of the logistic regression.

## Results

### Sample characteristics

A total of 358 adults were surveyed. The characteristics of the respondents are shown in Table 1. The mean age was 64.03 years (SD = 12.8), and most were aged 40–59 years (43.6%). A higher proportion of respondents were women (55.9%). Only 31.8% participants had an education level above high school. A higher proportion of the respondents had a monthly income less than the average city level (54.2%). Around half of the participants (45.5%) reported having contact with someone who had dementia, and around 80% of the participants had memory complaint.

**TABLE 1:** The characteristics of the respondents (*N* = 358).

Variables	*N* = 358
Age (mean ± SD)	64.0 ± 12.8
**Age groups**	
40–59	43.6%
60–69	24.0%
≥ 70	32.4%
**Gender**	
Male	158 (44.1%)
Female	200 (55.9%)
**Marital status**	
Married	262 (73.2%)
Single	96 (26.8%)
**Education level**	
Below high school	244 (68.2%)
High school and above	114 (31.8%)
**Monthly income^a^**	
Less than 4,000 CNY	194 (54.2%)
Above 4,000 CNY	164 (45.8%)
**Having a close relative with dementia**	
Yes	163 (45.5%)
No	195 (54.5%)
**Having memory complaint**	
Yes	286 (79.9%)
No	72 (20.1%)

^a^1 US dollar = 7 CNY.

### Prevalence of beliefs and knowledge about dementia prevention

**FIGURE 1:**
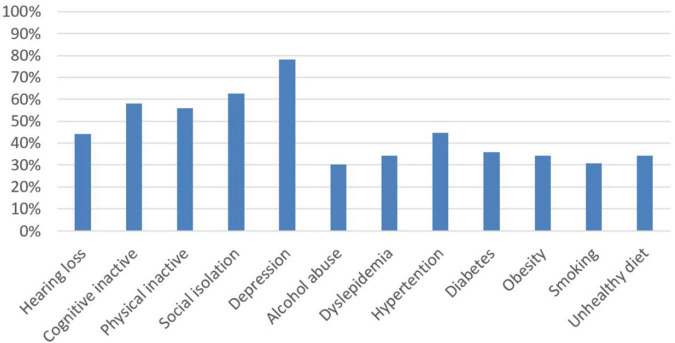
Proportion of participants who identified each dementia risk factor.

Approximately 41.9% of the respondents agreed that dementia is caused by normal aging. Less than one third of the respondents (32.4%) agreed that dementia could be preventable. The proportions of each item identified by the participants as a risk factor for dementia are presented in Figure 1. Most of the participants (99.2%) correctly identified at least one risk factor, but only 12.3% of the respondents identified all the risk factors correctly. Among the risk factors, “stress and depression” was endorsed by the most respondents (78.2%). The percentage of participants who accurately identified the risk factors of dementia was 62.6% for social isolation, 58.1% for cognitive inactivity, 55.9% for physical inactivity, 44.7% for hypertension, 44.1% for hearing loss, 36.3% for obesity, 35.8% for diabetes, 34.1% for dyslipidemia, 33.2% for unhealthy diet, 30.7% for smoking, and 30.2% for alcohol.

### Multivariable analysis of factors associated with dementia prevention beliefs

Table 2 presents the results for the significant correlates of dementia prevention beliefs. Compared with middle-aged adults (aged 40–59 years), older adults aged over 70 were more likely to believe that dementia is caused by normal aging (AOR = 1.99, 95% CI = 1.19–3.34) but were less likely to believe that dementia can be preventable (AOR = 0.47, 95% CI = 0.26–0.86). The participants with higher education level (high school and above) were more likely to believe that dementia can be preventable (AOR = 2.24, 95% CI = 1.34–2.75) than those with lower education level (below high school). Compared with the participants who were in contact with someone with dementia, the participants who were never in contact with patients with dementia were less likely to believe that dementia can be preventable (AOR = 0.48, 95% CI = 0.31–0.75). The beliefs of dementia prevention were not influenced by gender, income, marital status, or the presence of memory complaint.

**TABLE 2:** Socio-demographic factors associated with dementia prevention beliefs.

	Dementia is caused by normal aging		Dementia can be preventable	
	AOR	95% CI	AOR	95% CI
**Age group**				
40–59 (Ref)				
60–69	0.87	0.49–1.54	1.73	0.98–3.04
≥ 70	1.99*	1.19–3.34	0.47*	0.26–0.86
**Gender**				
Men (Ref)				
Women	0.99	0.63–1.57	1.35	0.83–2.20
**Income**				
< 4,000 CNY^a^ (Ref)				
≥ 4,000	1.08	0.67–1.74	1.03	0.62–1.72
Education				
Below high school (Ref)				
High school and above	0.56	0.34–0.93	2.24*	1.34–2.75
**Marriage**				
Single (Ref)				
Married	0.88	0.50–1.55	1.05	0.56–1.97
Memory complaint				
Yes (Ref)				
No	0.71	0.38–1.34	0.55	0.29–1.05
**Contact with dementia**				
Yes (Ref)				
No	1.22	0.76–1.99	0.48*	0.31–0.75

^a^1 US dollar = 6.5 CNY. *Significant results after Bonferroni correction for two measurements per subject (*P* < 0.05/2 = 0.025).

AOR, adjusted odds ratio; Ref, reference.

### Multivariable analysis of factors associated with knowledge on dementia risk factors

Table 3 present the results for the significant correlates of knowledge on dementia risk factors. Compared with middle-aged adults (aged 40–59 years), older adults were more aware that cognitive inactivity (aged ≥ 70 years: AOR = 3.84, 95% CI = 2.10–7.03), alcohol abuse (aged 60–69 years: AOR = 2.64, 95% CI = 1.49–4.66), dyslipidemia (aged ≥ 70 years: AOR = 2.48, 95% CI = 1.33–4.64), and unhealthy diet (aged ≥ 70 years: AOR = 2.73, 95% CI = 1.45–5.13) are dementia risk factors. Compared with the participants with lower education level (below high school), the participants with higher education level (high school and above) had a better understanding that obesity (AOR = 3.21, 95% CI = 1.79–5.75), smoking (AOR = 3.37, 95% CI = 1.79–6.34), and unhealthy diet (AOR = 3.57, 95% CI = 1.85–6.89) are risk factors for dementia. Compared with the participants who were in contact with someone with dementia, the participants who were never in contact with patients with dementia had lesser knowledge that diabetes (AOR = 0.41, 95% CI = 0.26–0.66) is a dementia risk factor. Gender, income, marital status, and the existence of memory complaint did not influence the awareness of dementia risk factors.

**TABLE 3:** Socio-demographic factors associated with knowledge of dementia risk factors.

	Hearing loss	Cognitive inactive	Physical inactive	Social isolation	Depression	Alcohol	Dyslipidemia	Hypertension	Diabetes	Obesity	Smoking	Unhealthy diet

	**AOR**	**AOR**	**AOR**	**AOR**	**AOR**	**AOR**	**AOR**	**AOR**	**AOR**	**AOR**	**AOR**	**AOR**
**Age group**												
40–59 (Ref)												
60–69	2.79	1.18	0.96	2.46	0.88	2.63*	1.04	1.06	1.05	0.83	2.48	1.13
≥ 70	1.96	3.84*	1.37	1.95	0.68	1.04	2.48*	1.59	1.25	2.32	0.96	2.73*
**Gender**												
Men (Ref)												
Women	1.23	1.01	1.30	1.26	1.59	0.97	0.97	1.29	1.08	1.09	0.82	1.14
**Income**												
< 4,000 CNY (Ref)												
≥ 4,000 CNY	1.94	0.93	1.66	0.80	1.51	1.58	1.58	1.16	1.35	1.33	1.79	1.51
**Education**												
Below high school (Ref)												
High school and above	1.76	2.02	0.77	0.70	0.67	1.09	1.09	2.10	1.35	3.21*	3.37*	3.67*
**Marriage**												
Single (Ref)												
Married	1.23	1.03	1.45	1.02	1.03	1.34	1.35	0.90	1.05	0.63	1.36	1.33
**Memory complaint**												
Yes (Ref)												
No	0.44	1.58	1.58	0.64	1.42	1.55	0.55	0.88	0.97	0.40	0.52	1.20
**Contact with dementia**												
Yes (Ref)												
No	0.98	1.18	1.05	0.66	0.46	1.04	1.04	0.65	0.41*	0.63	1.03	0.51

^a^1 US dollar = 6.5 CNY. *Significant results after Bonferroni correction for 12 measurements per subject (*P* < 0.05/12 = 0.004). AOR, adjusted odds ratio; Ref, reference.

### Health education needs of dementia

Most respondents (88.9%) thought that they were not well informed of dementia from public education by the government, media, or medical institution. When asked about their preferred health education delivery format, most respondents would like to receive advice from family physicians and community nurses (65%), followed by education booklets (60.9%), community bulletin (53.4%), health talks by experts (45.5%), and regular peer sharing (36.6%).

## Discussion

Latest evidence on the modifiable risk factors of dementia provides a strong rationale for focusing on dementia prevention by reducing dementia risk factors. The success of such efforts relies on the public’s understanding of dementia. The results of the survey revealed that, although huge scientific progress has been made on understanding the risk factors of dementia, a great gap still exists between the Chinese general public’s understanding of dementia prevention and contemporary scientific evidence. Additionally, beliefs on dementia prevention and an understanding of dementia risk factors are remarkably associated with socio-demographic variables, such as age, education level, and having contact with patients with dementia. These findings strongly suggest an urgent need to promote dementia prevention knowledge among the Chinese general public and develop different education strategies for people with different socio-demographic backgrounds.

Existing surveys of the general population on dementia prevention were predominantly conducted in Europe, the USA, and Australia. The results of the present study showed that 42.5% of our sample believed in the misconception that dementia is caused by normal aging and only 32.4% of them believed that dementia could be preventable. These proportions are even higher compared with those found in high-income countries, in which the survey results showed that 14–40% of the general public agreed that dementia is a part of normal aging (McParland et al., 2012; Tan et al., 2012; Seo et al., 2015) and 45–59% believed that dementia is not preventable (Connell et al., 2007; Smith et al., 2014; Seo et al., 2015). This misconception may delay seeking professional help and taking health behaviors to reduce dementia risk factors. Overall, the knowledge level of the risk factors of dementia among the Chinese general public is poor. Among all the dementia risk factors, social and psychological risk factors were endorsed more by the respondents, and cardiovascular risk factors were less endorsed as dementia risk factors. Previous studies also found that people have less awareness of the role of cardiovascular disease management in the development of dementia (Smith et al., 2014; Heger et al., 2019). This may be due the fact that dementia is predominantly regarded as a mental illness by the public, and there is lack of health education for the public to connect cardiovascular diseases with dementia. This finding highlights the need to design health education programs that emphasize the important link between cardiovascular risk factors and dementia.

The findings of this study regarding the factors associated with the beliefs of dementia prevention are consistent with previous studies (Smith et al., 2014; Seo et al., 2015). People with older age, a low level of education, and no contact with dementia were less likely to believe that dementia can be preventable. This study identified that participants with younger age are less knowledgeable about dementia risk factors. This finding is the opposite of another web-based study conducted in Asia (Zheng et al., 2020) but is consistent with the population-based survey conducted in Australia (Garvey et al., 2011). The inconsistency may be due to the different sampling methods across studies. The web-based study included young and highly educated participants, which may limit the representatives of the samples. The finding that younger participants are positive about dementia prevention but know less about dementia risk factors may be due to the fact that the younger generation is less concerned about the onset of dementia compared with older adults. Many of the dementia risk factors, such as cardiovascular and lifestyle risk factors, are likely different at all life stages; thus, public health programs on dementia prevention education should be provided across all age groups. The effects of dementia prevention strategies can be maximized this way. Lower education level was also identified to be associated with a lower understanding of dementia risk factors. This finding is in agreement with several previous studies (Low and Anstey, 2009; Roberts et al., 2014). This may because this disadvantaged group has limited access to the dementia prevention information, which highlights that more attention and resources should be invested in this group. Participants who have contact with patients with dementia were more knowledgeable of dementia risk factors probably because the situation renders oneself to pay more attention to the knowledge of dementia.

The majority of the respondents thought that they were not well informed of dementia from public education. Thus, the government, media, and medical institutions, as well as the communities, to make efforts to better popularize knowledge of dementia. In addition, receiving advice from family physicians and health education from community nurses is the preferred health education delivery format of the participants. This finding highlights the important role of primary care providers in dementia prevention. Ongoing education on dementia prevention for primary care providers is needed to transfer the latest scientific evidence to primary care practice.

Our study has several limitations that may influence the interpretation of the results. First, the conclusion of causality cannot be drawn because of the limitations of the cross-sectional study design. A repeated measures design would be ideal to track the trends in dementia prevention knowledge and its predictors. Second, in this study, only a limited number of communities were approached in a non-random manner for participants recruitment, thus selection bias may exist. Future studies should include a representative sample to increase the generalizability of the findings. Lastly, this study only adopted a quantitative survey study to investigate the research questions. Future studies are recommended to use in-depth qualitative approaches as a supplement to enrich our understanding on the public’s attitudes toward dementia prevention and its related factors.

## Conclusion

The present study reveals the great gaps between the Chinese general public’s knowledge of dementia prevention and the latest research evidence. The findings highlight the need for greater public awareness of dementia prevention and enable the promotion of culturally appropriate strategies to increase public awareness. Specifically, cardiovascular factors are the least endorsed by the public; hence, health education programs should emphasize the important link between cardiovascular risk factors and dementia. Special attention should be paid to the population with low income and low education, because they are associated with a low level of dementia prevention knowledge. We suggest this health advice could be delivered by primary care providers during routine chronic disease management, as we identified that the Chinese general public prefer receiving dementia-related health advice from family physicians and community nurses. The partnership between researchers and practitioners can help translate evidence into community practice in a timely manner.

## Data availability statement

The raw data supporting the conclusions of this article will be made available by the authors, without undue reservation.

## Ethics statement

The studies involving human participants were reviewed and approved by the Zhejiang Chinese Medical University (No. SBREC-20181231). The patients/participants provided their written informed consent to participate in this study.

## Author contributions

DS: conception and design of the work, acquisition, analysis, interpretation of the data, and drafted the work. DY and QHS: design of the work, acquisition, analysis, and interpretation of the data, and revised the work. All authors read and approved the final manuscript.
