# Supplementary material for: Perception and knowledge of dementia prevention and its associated socio-demographic factors in China: A community-based cross-sectional study
Source: Front Neurosci. 2022 Dec 5;16:1093169. doi: 10.3389/fnins.2022.1093169 (PMC9760739; doi:10.3389/fnins.2022.1093169)
Supplement: Supplementary file 1 [file Data_Sheet_1.docx]

**Questionnaire on the beliefs of dementia prevention, risk factors for dementia, and health education needs regarding dementia.**

1. **Beliefs of dementia prevention**

Please indicate your answer to the following questions by circling 1 of 3 options.

| Questions | Yes | No | No idea |
| --- | --- | --- | --- |
| Do you think that dementia is caused by normal aging? |  |  |  |
| Do you think that the risk of dementia can be reduced? |  |  |  |

1. **Risk factors for dementia**

Please indicate your answer to the following questions by circling 1 of 3 options (could be multiple choices).

| Items | Yes | No | No idea |
| --- | --- | --- | --- |
| Hearing loss |  |  |  |
| smoking |  |  |  |
| Alcohol abuse |  |  |  |
| Unbalanced and unhealthy diet |  |  |  |
| Hypertension |  |  |  |
| Diabetes |  |  |  |
| Dyslipidemia |  |  |  |
| Obesity |  |  |  |
| Physical inactivity |  |  |  |
| Cognitive inactivity |  |  |  |
| Depression |  |  |  |
| Social isolation |  |  |  |

1. **Health education needs regarding dementia prevention**

Please indicate your answer to the following questions by circling 1 of 3 options (could be multiple choices, except the first item).

| Items | Yes | No | No idea |
| --- | --- | --- | --- |
| 1. Whether you are well informed of dementia prevention from public education? |  |  |  |
| 1. Which dementia health education delivery format do you prefer？ |  |  |  |
| Community bulletin board |  |  |  |
| Health talks by experts |  |  |  |
| Advice from family physicians and community nurses |  |  |  |
| Education booklets |  |  |  |
| Regular peer sharing |  |  |  |
